# Chromosome aberrations and *HEY1-NCOA2* fusion gene in a mesenchymal chondrosarcoma

**DOI:** 10.3892/or.2014.3180

**Published:** 2014-05-15

**Authors:** IOANNIS PANAGOPOULOS, LUDMILA GORUNOVA, BODIL BJERKEHAGEN, KJETIL BOYE, SVERRE HEIM

**Affiliations:** 1 Section for Cancer Cytogenetics, Institute for Cancer Genetics and Informatics, The Norwegian Radium Hospital, Oslo University Hospital, Oslo, Norway; 2 Centre for Cancer Biomedicine, Faculty of Medicine, University of Oslo, Oslo, Norway; 3 Department of Pathology, The Norwegian Radium Hospital, Oslo University Hospital, Oslo, Norway; 4 Department of Oncology, The Norwegian Radium Hospital, Oslo University Hospital, Oslo, Norway; 5 Department of Tumor Biology, Institute for Cancer Research, The Norwegian Radium Hospital, Oslo University Hospital, Oslo, Norway; 6 Faculty of Medicine, University of Oslo, Oslo, Norway

**Keywords:** mesenchymal chondrosarcoma, cytogenetics, fusion gene, *HEY1-NCOA2*

## Abstract

Mesenchymal chondrosarcomas are fast-growing tumors that account for 2–10% of primary chondrosarcomas. Cytogenetic information is restricted to 12 cases that did not show a specific aberration pattern. Recently, two fusion genes were described in mesenchymal chondrosarcomas: a recurrent *HEY1-NCOA2* found in tumors that had not been cytogenetically characterized and an *IRF2BP2-CDX1* found in a tumor carrying a t(1;5)(q42;q32) translocation as the sole chromosomal abnormality. Here, we present the cytogenetic and molecular genetic analysis of a mesenchymal chondrosarcoma in which the patient had two histologically indistinguishable tumor lesions, one in the neck and one in the thigh. An abnormal clone with the G-banding karyotype 46,XX,add(6)(q23),add(8)(p23),del(10)(p11),+12,−15[6] was found in the neck tumor whereas a normal karyotype, 46,XX, was found in the tumor of the thigh. RT-PCR and Sanger sequencing showed that exon 4 of *HEY1* was fused to exon 13 of *NCOA2* in the sample from the thigh lesion; we did not have spare material to perform a similar analysis of the neck tumor. Examining the published karyotypes we observed numerical or structural aberrations of chromosome 8 in the majority of the karyotyped mesenchymal chondrosarcomas. Chromosome 8 was also structurally affected in the present study. The pathogenetic mechanisms behind this nonrandom involvement are unknown, but the presence on 8q of two genes, *HEY1* and *NCOA2*, now known to be involved in mesenchymal chondrosarcoma tumorigenesis is, of course, suggestive.

## Introduction

Mesenchymal chondrosarcomas are rare tumors that account for 2–10% of primary chondrosarcomas (1). Their typical histological appearance includes a biphasic pattern with areas of round, primitive mesenchymal cells and interspersed islands of well differentiated hyaline cartilage (2). They are two to three times more common in bone than in soft tissue and are mostly found in the head and neck area, particularly the orbit, the cranial and spinal dura mater, and the lower extremities, especially the thigh (2). However, rare cases of mesenchymal chondrosarcoma have been described in virtually every anatomic site. Unlike other types of chondrosarcoma, mesenchymal chondrosarcomas grow fast and often give rise to local recurrences and metastases. The majority of the cases are diagnosed in the second and third decade of life and the prognosis is highly variable with published 10-year overall survival rates ranging from 21% to 67% (1). Moreover, some patients live for long periods with metastatic disease, whereas others die shortly after diagnosis (1). Adequate surgery is the treatment of choice for localized disease (3). The role of chemotherapy and radiotherapy remains poorly defined (4,5).

To date, only 12 mesenchymal chondrosarcomas have been karyotyped (6–15) and no consistent aberration pattern has been established. Recently, however, two fusion genes were reported in mesenchymal chondrosarcomas. Wang *et al* used a genome-wide exon-resolution expression screen to identify a fusion between the hairy/enhancer-of-split related with YRPW motif 1 (*HEY1*; on 8q21.13) gene and the nuclear receptor coactivator 2 (*NCOA2*; on 8q13.3) gene; no karyotypic data were available on the tumors thus examined (16). Nyquist *et al* used karyotyping followed by RNA-Seq to identify an *IRF2BP2-CDX1* fusion gene in a case of mesenchymal chondrosarcoma carrying a solitary t(1;5)(q42;q32) chromosomal translocation (12). Here, we present a mesenchymal chondrosarcoma which proved to have an informative karyotype and which, by RT-PCR, was found to carry a *HEY1*-*NCOA2* fusion gene.

## Materials and methods

### Ethics statement

The study was approved by the regional ethics committee (Regional komité for medisinsk forskningsetikk Sør-Øst, Norge, http://helseforskning.etikkom.no) and written informed consent was obtained from the patient.

### Case report - pathology

**Figure 1:**
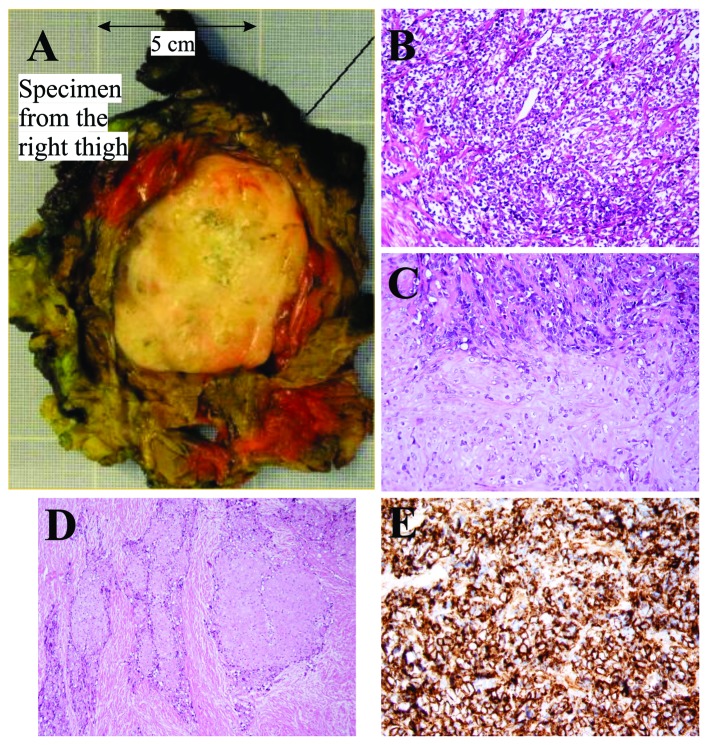
Pathological features of the mesenchymal chondrosarcoma from the thigh. (A) Macroscopic image of the tumor from the right thigh. (B) Areas with small round cells. (C and D) Biphasic growth pattern showing chondroid areas with transition to small round cells. (E) Positive immunohistochemical reaction of CD99.

The patient was a 26-year-old woman who had noticed a tumor in the right thigh three months prior to diagnosis. Radiologic evaluation revealed a 9.0×8.0×5.4 cm large tumor in the large adductor muscle. A soft tissue lesion, presumed to be a metastasis, was detected in the neck, and another lesion of unknown origin was found in the left iliac bone. The patient received four cycles of chemotherapy; two cycles of vincristine, doxorubicin and cyclophosphamide, one cycle of vincristine, ifosfamide and actinomycin D, and one cycle of etoposide and ifosfamide. Radiologic evaluation after chemotherapy revealed no significant change in size of any of the tumors. A wide resection of the primary tumor (thigh) was performed (Fig. 1A). Radiotherapy and subsequent surgery of the metastasis in the neck is planned.

Microscopic examination of the specimen from the thigh showed a biphasic tumor with cellular areas with high-grade, malignant-looking, small undifferentiated round cells and some more pleomorphic cells (Fig. 1B) alternating with cartilage of hyaline type consistent with a low-grade malignant chondrosarcoma (Fig. 1C). The transition between the two components was mostly abrupt. There was bone formation close to the chondroid areas. A desmoplastic stroma was seen both around the small round cells and the chondroid areas (Fig. 1D). Immunohistochemical analysis showed a strong positive reaction to the antibody CD99 in the small round cells (Fig. 1E). Microscopic examination of the lesions in the neck and iliac bone showed tumor tissue consistent with mesenchymal chondrosarcoma.

### Karyotyping

Tumor samples from both the neck and thigh were removed by core needle biopsy. The samples were mechanically and enzymatically disaggregated and then short-term cultured as described elsewhere (17). The cultures were harvested and the chromosomes G-banded using Wright stain. The subsequent cytogenetic analysis and karyotype description followed the recommendations of the ISCN (18).

### Reverse transcription-polymerase chain reaction (RT-PCR)

Total RNA was extracted using TRIzol reagent from the core needle biopsy of the tumor of the thigh. No material was available from the tumor of the neck. Then, 1 μg of total RNA was reverse-transcribed in a 20 μl reaction volume using iScript Advanced cDNA Synthesis Kit for RT-qPCR according to the manufacturer’s instructions (Bio-Rad). The cDNA was diluted to 50 μl and 2 μl (corresponding to 40 ng of total RNA) were used as template in subsequent PCR assays. As a positive control, a mesenchymal chondrosarcoma known to carry the *HEY1-NCOA2* fusion was used (12). The 25 μl PCR-volume contained 12.5 μl of Premix Taq (Takara Bio Europe/SAS, Saint-Germain-en-Laye, France), 2 μl of diluted cDNA, and 0.2 μM of each of the forward HEY1-F1 (CGAGGTGGAGAAGGAGAGTG) and reverse NCOA2-E13-R3 (AGTTGGGCTTTGCAATGTGA) primers. Both samples were tested for expression of the *HEY1* gene to assess the quality of RNA and cDNA synthesis. The PCR components were the same as above except that the primer combination HEY1-F1 and HEY1-551R (CTCCGATAGTCCATAGCAAGG) was used. The PCRs were run on a C-1000 Thermal Cycler (Bio-Rad) using the following cycling conditions: an initial denaturation at 94°C for 30 sec followed by 35 cycles of 7 sec at 98°C, 30 sec at 55°C and 2 min at 68°C, and a final extension for 5 min at 68°C.

Four microliters of the PCR products were stained with GelRed (Biotium, Hayward, CA, USA), analyzed by electrophoresis through 1.0% agarose gel, and photographed. The amplified fragment was purified using the Qiagen gel extraction kit (Qiagen). Direct (Sanger) sequencing was performed using the light run sequencing service of GATC Biotech (http://www.gatc-biotech.com/en/sanger-services/lightrun-sequencing.html). The BLAST software (http://www.ncbi.nlm.nih.gov/BLAST/) was used for computer analysis of sequence data.

## Results

**Figure 2:**
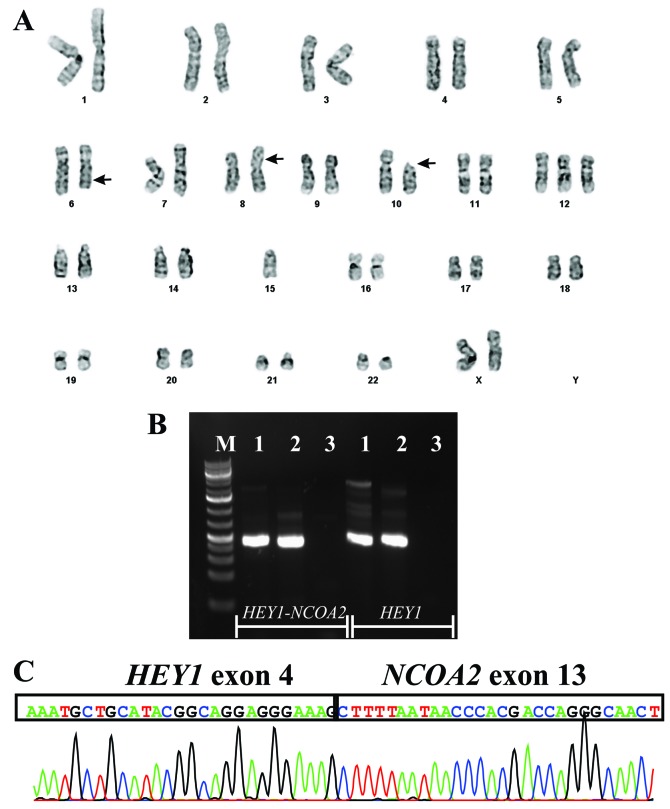
Cytogenetic and PCR analyses of the mesenchymal chondrosarcoma. (A) Karyotype of the tumor of the neck; breakpoint positions are indicated by arrows. (B) RT-PCR of RNA extracted from the tumor of the thigh. cDNA fragment amplifications of *HEY1-NCOA2* using primers HEY1-F1 and NCOA2-E13-R3 and normal *HEY1* with primers HEY1-F1 and HEY1-551R. M, 1 Kb Plus DNA ladder (GeneRuler, Fermentas). (C) Partial sequence chromatogram showing that exon 4 of *HEY1* is fused to exon 13 of *NCOA2*.

The G-banding analysis of cells cultured from the tumor of the neck yielded the karyotype 46,XX,add(6)(q23),add(8)(p23),del(10)(p11),+12, −15[6]/46,XX[5] (Fig. 2A), whereas a normal karyotype, 46,XX, was found in the tumor of the thigh.

RT-PCR with the HEY1-F1/NCOA2-E13-R3 primer combination amplified a single cDNA fragment in both the tumor of the thigh and the positive control (Fig. 2B). Sequencing of the amplified fragment showed that exon 4 of *HEY1*(nt 531 in sequence with accession number NM_012258 version 3) was fused to exon 13 of NCOA2 (nt 2768 in sequence with accession number NM_006540 version 2) (Fig. 2C). Normal *HEY1*cDNA fragments were amplified in both cases (Fig. 2B).

## Discussion

Despite being a recognized entity for more than 50 years, mesenchymal chondrosarcoma continues to present substantial diagnostic, prognostic and management challenges, due, in large part, to its rarity (1). The cytogenetic information is restricted to 12 cases with variable karyotypes and no consistent aberration pattern has been established. Dobin *et al* reported an intrathoracal mesenchymal chondrosarcoma with a near-tetraploid karyotype which included structural chromosomal abnormalities such as add(7)(p13), add(22)(q13), markers and double minutes (7). Gatter *et al* reported trisomy 8 as the sole cytogenetic abnormality (9), while three other reports described different chromosomal translocations, t(4;19)(q35;q13), t(6;10)(p21;q22), and t(1;5)(q42;q32), as the sole cytogenetic abnormality (6,12,13). Nevertheless, examining the karyotypes as part of this study we observed that involvement of chromosome 8 was found in as many as 7 out of the 12 karyotyped mesenchymal chondrosarcomas with aberrations: +8 was found in three cases (7,9,14), −8 was found in two cases (8,10), whereas structural aberrations of 8q were found in three cases (in one case together with −8) (8,11). Chromosome 8 was also structurally affected in the present study. The pathogenetic mechanisms behind this nonrandom involvement are unknown, but the presence on 8q of two genes, *HEY1* and *NCOA2*, now known to be involved in mesenchymal chondrosarcoma tumorigenesis is, of course, suggestive.

Recently, Wang *et al* identified a *HEY1-NCOA2* fusion in mesenchymal chondrosarcomas. Using a combination of FISH and RT-PCR methodologies they found that 10 out of 15 examined mesenchymal chondrosarcomas carried the *HEY1-NCOA2* (16). The findings were verified in two other studies: Nyquist *et al* (12) showed by RT-PCR the presence of *HEY1-NCOA2* in 3 of 4 mesenchymal chondrosarcomas, and Nakayama *et al* (19) found *HEY1-NCOA2* in 8 of 10 mesenchymal chondrosarcomas using FISH on formalin-fixed and paraffin-embedded samples. The absence of *HEY1-NCOA2* fusion in some cases has been explained as being due to methodological inadequacy (16,19) but the possibility of other disease-specific fusion gene(s), and thus pathogenetic heterogeneity in this diagnostic entity, should not be ruled out. As demonstrated by Nyquist *et al*, the chromosomal translocation t(1;5)(q42;q32) resulted in fusion of *IRF2BP2* (located on 1q42) with *CDX1* (on 5q32) to generate an *IRF2BP2-CDX1* fusion gene in the mesenchymal chondrosarcoma they studied (12). In spite of this, the *HEY1-NCOA2* fusion does seem to be common as well as specific for mesenchymal chondrosarcomas since it was not found in conventional and dedifferentiated chondrosarcomas (16). Both genes are located on the long arm of chromosome 8, *HEY1* in 8q21.13 and *NCOA2* in 8q13.3, therefore the fusion may result from an interstitial deletion (16) or a t(8;8)(q13;q21) chromosomal translocation.

The involvement of the *NCOA2* gene in neoplasia was first reported by Carapeti *et al* who showed that in acute myeloid leukemia the cytogenetic aberration inv(8)(p11q13) resulted in a *KAT6A-NCOA2*, also known as *MOZ-TIF2*, fusion gene (20,21). Since then, the gene has also been implicated in various other malignancies. Strehl *et al* identified a novel recurrent t(8;12)(q13;p13) resulting in a fusion between the transcriptional repressor *ETV6 (TEL)* and *NCOA2* in six cases of childhood leukemia expressing both T-lymphoid and myeloid antigens (22). A *PAX3-NCOA2* gene was found as a rare variant fusion in alveolar rhabdomyosarcoma; it was brought about by a t(2;8)(q35;q13) translocation (23). The *AHRR-NCOA2* and *GTF2I-NCOA2* fusion genes were described in soft tissue angiofibroma through translocations t(5;8)(p15;q13) and t(7;8)(q11;q13), respectively, emphasizing the role of *NCOA2* in soft tissue angiofibroma development (24,25). Recently, *SRF-NCOA2* and *TEAD1-NCOA2* fusions were reported in rhabdomyosarcomas (26). In all the above mentioned fusions, *NCOA2* is the 3′-partner gene and all fusion proteins contain the two C-terminal activation domains AD1/CID (activation domain 1/CREB binding protein interacting domain) and AD2 (20–26). The transforming activities of *KAT6A-NCOA2* and *PAX3-NCOA2* have been demonstrated experimentally (23,27). In addition, KAT6A-NCOA2 was shown to induce acute myeloid leukemia in transgenic fish (28). Deguchi *et al* (27) showed that the KAT6A-NCOA2 interaction with CREBBP through AD1/CID is essential for transformation. Similarly, Sumegi *et al* (23) showed that while deletion of the AD2 portion of PAX3-NCOA2 fusion protein reduced transforming activity, deletion of the AD1/CID domain fully abrogated the transforming activity of the chimeric protein. Thus, the presence of the AD1/CID and AD2 domains of NCOA2 seems to be essential for the transformation capacity of the various cancer fusion genes.

*HEY1* encodes a nuclear protein belonging to the hairy and enhancer of split-related (HESR) family of basic helix-loop-helix (bHLH)-type transcriptional repressors (29). Expression of this gene is induced by the Notch and c-Jun signal transduction pathways (30). HEY1 protein binds to specific DNA sequences in the promoter regions of target genes as a dimer, recruiting co-repressors to repress the target genes of Notch signaling. The HEY1-NCOA2 fusion replaces the C-terminal portion of HEY1 by the NCOA2 AD1/CID and AD2 domains, while retaining the HEY1 bHLH DNA-binding/dimerization domain. Therefore, the HEY1-NCOA2 fusion protein, instead of recruiting co-repressors, may recruit co-activators through its NCOA2 part to some Notch/HEY1 target genes (16). Additional experiments are required to confirm or falsify the validity of this hypothesis.
